# Structural and Mechanical Changes of AlMgSi_0.5_ Alloy during Extrusion by ECAP Method

**DOI:** 10.3390/ma15062020

**Published:** 2022-03-09

**Authors:** Marta Harničárová, Jan Valíček, Milena Kušnerová, Ivan Kopal, Miloslav Lupták, Rastislav Mikuš, Zdeněk Pavelek, Martin Fabián, Vladimír Šepelák

**Affiliations:** 1 Department of Mechanical Engineering, Faculty of Technology, Institute of Technology and Business in České Budějovice, Okružní 10, 370 01 České Budějovice, Czech Republic; valicek.jan@mail.vstecb.cz (J.V.); kusnerova.milena@mail.vstecb.cz (M.K.); 28213@mail.vstecb.cz (Z.P.); vladimir.sepelak@kit.edu (V.Š.); 2 Institute of Electrical Engineering, Automation, Informatics and Physics, Faculty of Engineering, Slovak University of Agriculture in Nitra, Tr. A. Hlinku 2, 949 76 Nitra, Slovakia; 3 Department of Numerical Methods and Computational Modeling, Faculty of Industrial Technologies in Púchov, Alexander Dubček University of Trenčín, Ivana Krasku 491/30, 020 01 Púchov, Slovakia; ivan.kopal@tnuni.sk; 4 Institute of Materials and Quality engineering, Faculty of Materials, Metallurgy and Recycling, Technical University of Košice, Letná 9, 042 00 Košice, Slovakia; miloslav.luptak@tuke.sk; 5 Institute of Design and Engineering Technologies, Faculty of Engineering, Slovak University of Agriculture in Nitra, Tr. A. Hlinku 2, 949 76 Nitra, Slovakia; rastislav.mikus@uniag.sk; 6 Institute of Geotechnics, Slovak Academy of Sciences, 040 01 Košice, Slovakia; fabianm@saske.sk; 7 Institute of Nanotechnology, Karlsruhe Institute of Technology (KIT), Hermann-von-Helmholtz-Platz 1, 76344 Eggenstein-Leopoldshafen, Germany

**Keywords:** aluminium alloy, intensive plastic deformation method, microstructure, mechanical properties

## Abstract

SPD (several plastic deformations) methods make it possible to obtain an ultrafine-grained structure (UFG) in larger volumes of material and thus improve its mechanical properties. The presented work focuses on the structural and mechanical changes of aluminium alloy AlMgSi0.5 (EN AW 6060) during processing by repeated extrusion through the ECAP rectangular channel. After a four-pass extrusion, the samples’ microstructures were observed using an optical microscope, where refinement of the material grains was confirmed. Tensile tests determined the extrusion forces and allowed interpretation of the changes in the mechanical properties of the stressed alloy. The grain size was refined from 28.90 μm to 4.63 μm. A significant improvement in the strength of the material (by 45%) and a significant deterioration in ductility (to 60%) immediately after the first extrusion was confirmed. The third pass through the die appeared to be optimal for the chosen deformation path, while after the fourth pass, micro-cracks appeared, significantly reducing the strength of the material. Based on the measurement results, new analytical equations were formulated to predict the magnitude or intensity of the volumetric and shape deformations of the structural grain size and, in particular, the adequate increase in the strength and yield point of the material.

## 1. Introduction

Nowadays, the demand for more sophisticated materials is growing rapidly. Therefore, one of the objects of current research in the field of advanced materials is the continuous improvement of their design to improve their physical, chemical, and mechanical properties leading to their use in high-end applications. Modifying the microstructure of a material is an effective method of tailoring its properties to achieve specific improvements in material properties. In this paradigm, refining the grain size of crystalline materials to produce ultrafine-grained (UFG) or nanocrystalline (NC) materials has been shown to be an effective technique to increase their strength [1,2,3]. Such a refined microstructure can be achieved by processes involving severe plastic deformation (SPD) or compacting ultrafine powders or nanoparticle-sized powders from bulk materials using various powder metallurgy processes. Research in this area has been growing rapidly over the last decade, with the main objectives being to increase the productivity of the processes and to understand the deformation mechanisms of these materials better [4,5,6].

One of the most widely used methods of intensive plastic deformation to refine the microstructure of materials today is ECAP (Equal Channel Angular Pressing). The ECAP method can be applied to a wide variety of metals and alloys to create ultrafine grains and to improve mechanical and physical properties. Titanium, copper, aluminium, and aluminium alloys are the most commonly used materials in SPD-ECAP studies, with aluminium alloys finding applications mainly in the automotive, aerospace, and space industries due to their excellent mechanical properties, corrosion resistance, and higher strength-to-weight ratio. The development of the principles underlying the SPD techniques dates back to the 1930s to the pioneering work of P.W. Bridgman of Harvard University. This work concerned the effects of a combination of large hydrostatic pressures and simultaneous shear deformation acting on solids. The main objective of this process is to produce very strong and lightweight parts that would find applications in engineering practice [7,8]. The method of intense plastic deformation (SPD) is a general term describing a group of metal processing techniques. The method consists of the creation of a highly fragmented, inhomogeneous, anisotropic structure due to large deformations, which retains the residual features of the recrystallised amorphous state [1]. The purpose of these technologies is to obtain non-porous metals and alloys with an “ultrafine grain size” of *d* < 500 nm, or directly nanocrystalline structures (*d* < 100 nm) [9].

The grain size of polycrystalline materials affects many critical material properties, including strength and resistance to plastic deformation. Materials with a small grain size have several advantages over their coarse-grained counterparts, which include higher strength, an extremely high yield point, high hardness, improved toughness, and other favourable properties. Many traditional high-volume formation methods involving simple rolling, drawing, or extrusion cannot meet the requirements for the reliable forming of nanostructured materials. The formation of nanostructures in high-volume samples is quite challenging and often not possible except by using special mechanical deformation methods at low or elevated temperatures [9].

The ECAP method is used to obtain high-density nanostructured materials with high grain morphological uniformity from massive plastic deformable semi-finished products. Shear deformation of the sample occurs when it crosses the contact surface between the channels. After several repetitions of the ECAP procedure, a systematic increase in deformation occurs, leading to a gradual decrease in grain size due to the formation of a network of small-angle and then large-angle grain boundaries. This characteristic of the method makes it possible to subject ductile materials and difficult-to-form metals and alloys to intense plastic deformation. The ECAP technology can also be used to control the crystallographic structure of bulk structural materials [10].

The extrusion angle at which the mould channels intersect is the most important experimental factor affecting grain, as it determines the overall reshaping of the blank after each pass through the die. In most applications, an angle between 90° and 120° is used. This is because, despite the high efficiency of the ECAP process with a 90° refracted channel, in reality, it is much easier to push very hard materials and materials with low ductility through a die with an angle greater than 90° (e.g., 110°) [9]. In practice, the reshaping of the material after each extrusion process depends on both the extrusion angle between the two parts of the channel and the angle describing the outer arc of curvature at the point where the inlet and outlet channels intersect [7]. Calculations show that this angle plays only a minor role for channel angles of 90° and above. On the other hand, for channel angles less than 90° (e.g., 60°), this angle has a much more significant effect on material reshaping than the extrusion angle [10] in many experiments. By changing the orientation of the sample before re-extrusion, different microstructures and textures of the material can be created. Several processing methods have been developed to achieve deep grain refinement (in the order of nanometres) by changing the orientation after each pass. In order to obtain different textures and microstructures, three basic ECAP routes are defined, which are referred to as A, B, and C.

In procedure A, the orientation of the sample remains unchanged after each push. In procedure B, the sample is rotated 90° about its longitudinal axis after each pass. If the rotation is always in the same direction, the route is $B_{A}$. If the direction of rotation alternates counter-clockwise and clockwise, the route is $B_{C}$. It is procedure C if the sample is rotated 180° about its longitudinal axis after each pass. The difference between methods A, B, and C is, therefore, the direction of shear and the orientation of its plane. In a series of experiments, it was shown that procedure $B_{C}$ is a very good method of processing materials and producing ultrafine microstructures because it deforms the sample uniformly in all planes [7,9,11].

Similar to the temperature, speed, and orientation of the sample during extrusion, the number of passes plays an important role in determining the progress of recrystallisation and the resulting mechanical properties during the ECAP process. As mentioned above, curing of different alloys can be achieved by implementing different numbers of ECAP passes, which implies that the grain size, refinement intensity, relative elongation of the material, and structure are also greatly influenced by the number of die passes. For most alloys, although the grain refinement is continuous but less intensive as the number of passes increases, the ductility and yield point also decrease [7,9].

Polycrystalline materials are classified into several categories according to grain size. They can be classified as coarse-grained if the average grain size is greater than 10 µm, as fine-grained if the average grain size is from 10 to 0.5 µm, as ultrafine-grained if the average grain size is from 500 to 100 nm, and as nanocrystalline (NC-Nanocrystalline (Nanostructured) Materials) if the average grain size is from 100 to 1 nm [8,12]. The production of these materials is feasible in two ways. In the first approach, known as “bottom-up”, polycrystalline metals are assembled from single atoms using deposition techniques or from fabricated nanoscale building blocks, e.g., by ultrasonic milling or by compacting a pre-prepared nano powder [8]. However, these techniques have major drawbacks, as the products are very small and suitable for use mainly in microdevices and always contain at least a small amount of residual porosity and impurities from manufacturing [13]. The second option is the top-down approach, in which larger coarse-grained polycrystalline solids are processed by severe plastic deformation. The purpose is to refine their microstructure with minimal change in the initial dimensions of the workpiece. The resulting grain sizes are typically in the range of 100 to 1000 nm or, in some cases, less than 100 nm. UFG materials have been shown to exhibit excellent strength at room temperature and good superplastic properties at elevated temperatures. As a result of the larger dimensions, the products then have potential use in a variety of structural applications. The most important of these two methods is the “top-down” method, as it has enabled the development of the methods of severe plastic deformation (SPD) [8,13].

Many works have been published describing the results obtained in the formation of, mainly through ECAP, aluminium and its alloys, nickel, copper, and later, pure iron, steel, titanium, and other metals and alloys [14,15]. Several plastic deformation methods significantly change the mechanical properties of metals and their alloys, especially their strength and ductility. This has been demonstrated by several recent papers dealing with Al-Mg, Al-Mn, and also Al-Mn-Mg alloys and other difficult-to-work materials [16].

An important factor that significantly influences the microstructure development is the *Φ* angle, formed by the channels’ longitudinal axes. This angle determines the amount of shear deformation in the individual passes. A smaller *Φ* angle results in a higher shear deformation at each pass, and this arrangement is, therefore, more effective in grain refinement. The influence of the angle in the range from 90° to 157.5° was studied on aluminium by Nakashima et al. [17] using the technological path B_C_. They found that grain refinement is most effective, at the same volume of deformation, at an angle of 90°. They attributed this result to an angle of 60°, which is formed by two shear planes in the deformed billet. For more difficult-to-form materials (steels, Ti alloys), an angle of 120° and a higher extrusion temperature is usually used.

During the application of large deformations, the grain size gradually converges with the size of the subgrains because the subgrains have the smallest possible dimensions at which the grain boundaries can absorb dislocations. The finest structures can be achieved at low homologous temperatures and the highest strain rates. Grain size is also very sensitive to the presence of alloying elements that slow down healing, such as the alloying of aluminium with magnesium [18]. Wagner et al. [19] subjected AA6060 aluminium to ECAP and provided information about the formation of shear and matrix bands resulting in the strain partitioning with cracking. After performing SEM analysis, a structure similar to that formed in ultrafine grain materials was observed. Karon et al. [20] investigated the properties and microstructure of AN 6060 after ECAP. Again, an increase in strength properties and a decrease in ductility were observed. In addition to studying the impact of ECAP on the mechanical properties of aluminium alloys, Yulinova et al. [21] were focused on the electrochemical properties. These investigators did not observe a worsening of the electrochemical corrosion behaviour of the Al alloy after ECAP.

Chung et al. [22] studied the fatigue life of aluminium alloy 6061 subjected to ECAP. After one pass, the authors reported a significant increase in low- and high-cycle fatigue life. However, further deformation eliminated this improvement, especially in high-cycle fatigue. Ch. Xu et al. [23] investigated the superplastic behaviour of three aluminium alloys (Al-Mg-Sc, Al-Cu-Mg, and Al-Zn-Mg-Ca-Zr) after large plastic deformation by the ECAP method. After deformation, the grain size was in the range of 200–300 nm. All alloys showed a good to exceptional ability for superplastic forming (2000, 450, and 1100% elongation). Horita et al. [24] reported that the elongation at break was over 1500% after ECAP Al-Mg-Sc. Islamgaliev et al. [25] investigated the superplasticity characteristics of Al alloy 1421 (Al-Mg-Li-Zr-Sc) after ECAP (8 passes at 370 °C, achieved grain size—800 nm). At a strain rate of 10^−2^ s^−1^, they reached an elongation of over 1300%. Experimental results show that aluminium alloys subjected to ECAP have increased superplasticity at relatively low temperatures and high strain rates.

### Theoretical Background

Ultrafine-grained materials (UFG) are materials with a grain size below 1 μm. Their strength can be derived using the Hall–Petch relation (1):

(1) σy=σo+kxy⋅d−12

where $\sigma_{y}\;$is the yield point [MPa], $\sigma_{o}$ is the frictional stress required for the movement of dislocations, *k_xy_* is the material constant [N∙mm^−3/2^], and *d* is the mean grain diameter [mm]. From relation (1), it follows that the smaller the grain size, the stronger the material, and at the same time, the yield point increases. In some cases, however, softening of the material can also occur if the grains are reduced below a certain critical size.

A more detailed elucidation of the Hall–Petch relationship in the investigation of oxygen-free copper after the ECAP treatment was discussed by Meshal Y. Alawadhi et al. (2021) [26]. In their research, the yield stress was plotted as a function of the inverse of the square root of the average grain size. It was found that the resulting graph is definitely not linear over the entire range of grain sizes used in this research. In the range of grain sizes $d^{-1/2}<880\;m^{-1/2}$, the values are consistent with relation (2), but at smaller grain sizes (if $d^{-1/2}>880\;m^{-1/2}$), the constant $k_{y}$ reached negative values. These results indicate a transition from hardening to softening of the material and an inverse Hall–Petch effect with much larger values (at grain sizes of approximately less than 1 μm) than for the intermediate grain size transition (at grain sizes of approximately less than 2 nm).

Thus, two strengthening mechanisms contribute to the overall strength of oxygen-free copper: Grain boundary strengthening $\sigma_{GB}$ and dislocation strengthening $\sigma_{dis}$. The total stress is, therefore, a combination of these strengthening mechanisms with the assumption that each of them works independently (2):

(2) $$\sigma =\sigma_{o}+\sigma_{GB}+\sigma_{dis}$$

Grain boundary strengthening is based on the Hall–Petch relation (2), while dislocation strengthening is defined by the Taylor Equation (3) [27]:

(3) σdis=α⋅M⋅G⋅b⋅ρ12

where *α* is a numerical factor depending on the dislocation arrangement, *M* is a Taylor factor with a constant value of 3.06 for uniaxial tensile deformation in the fully constrained model, *G* is the shear modulus, *b* is the Burgers vector, and *ρ* is related to the density of dislocations. In practice, the dislocation density and yield stress generally show a similar evolution to the stress values when processed by the ECAP method. It can be seen that the yield stress at saturation is related to the maximum dislocation density by the Taylor relation for metals with a surface-centred crystal lattice (f.c.c.) [26]. The dislocation density increases while the grain size decreases with stress during the deformation process in the case of the ECAP technology. Thus, after combining the previous relations (1), (2), and (3), the total strength is (4):

(4) σ=σo+kxy⋅d−12+α⋅M⋅G⋅b⋅ρ12

On the basis of the above research, the main objective was set, namely, to investigate the relationship between the development of structural and mechanical changes of the AlMgSi_0.5_ alloy during extrusion by the ECAP method. When this knowledge is used at the design stage, the extrusion technology can be more effectively designed and controlled to achieve the desired grain size and material strengthening.

## 2. Materials and Methods

### 2.1. Experimental Material AlMgSi_0.5_

Aluminium alloy AlMgSi_0.5_ (EN AW 6060) is a medium-strength, heat-treatable alloy with a strength slightly below 6005 A. Alternative names and designations include Al-Mg-Si_0.5_, 3.3206, and A96060. It is a heat-treatable alloy with excellent corrosion resistance, weldability, and machinability.

It is also characterised by good cold formability. It is commonly used for the production of complex cross-sections, can be easily anodised, and is suitable for welding with all common methods. Its chemical composition according to standards and tolerances are listed in Table 1.

The test material samples were measured in the experiment through the ECAP process. The samples of the selected material EN AW 6060 were forced through a hydraulic press through a 90° angled channel. After their extrusion and evaluation of the forces, one sample from each pair was ground with different fine grinding wheels and polished in an electropolisher. Subsequently, after chemical etching, their microstructure was measured as a function of the number of passes through the die using a GX51 metallographic microscope. For the second sample in each pair, test bars with a square cross-section of non-normalised dimensions were fabricated, and their mechanical properties (yield point, ultimate tensile stress, ductility, modulus of elasticity, and Poisson’s number) were further analysed and recorded by tensile tests.

The use of the selected alloy is varied. It is most often applied to medium-strength components operating at temperatures of 50–70 °C for long periods of time, where good processability, corrosion resistance, or decorative appearance is required. Typical application areas are materials for furniture, decorative items, automotive finishes, light poles and masts, architecture, and the food industry.

EN AW 6060 is much more closely related to 6063 than 6061. The main difference between 6060 and 6063 is that 6063 has a slightly higher magnesium content. Applications requiring a higher-strength material typically use 6061 or 6082 aluminium alloy.

The starting material for testing intensive plastic deformation by ECAP was a pressed bar of a circular cross-section made of Al-Mg-Si_0.5_ (alloy EN AW 6060) with a diameter of 10 mm. It is a structural material with good ductility, gloss, and corrosion resistance. In addition, its already-mentioned applications include the aerospace and automotive industries, construction, and fine mechanics (e.g., aircraft and helicopter parts, car bodies, and measuring equipment components).

### 2.2. Experiment Preparation

**Figure 1:**
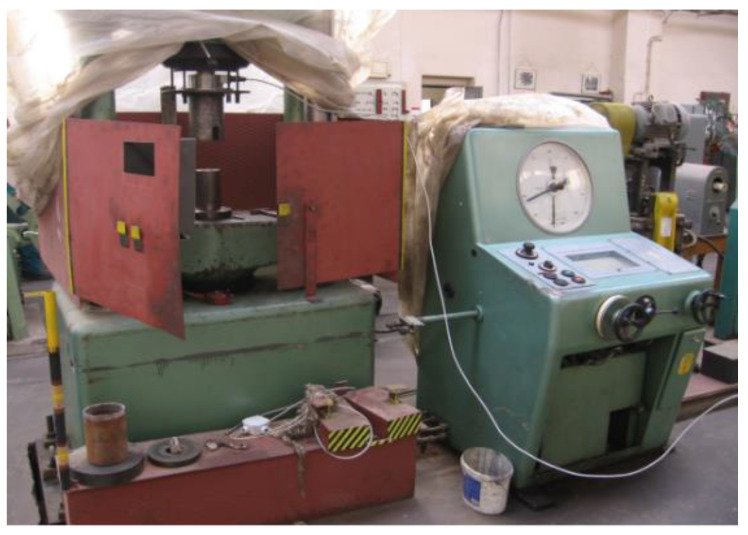
The hydraulic press used in the experiment.

The hydraulic press used is shown in Figure 1. The sample size was limited to a maximum length of 100 mm and a circular cross-section of 10 mm. These samples were processed through a total of four passes through route C (in procedure C, the sample is rotated 180° after each pass), where the channel angle was *Φ* = 90° and the external channel curvature angle *Ψ(R)* ≅ 0°.

**Figure 2:**
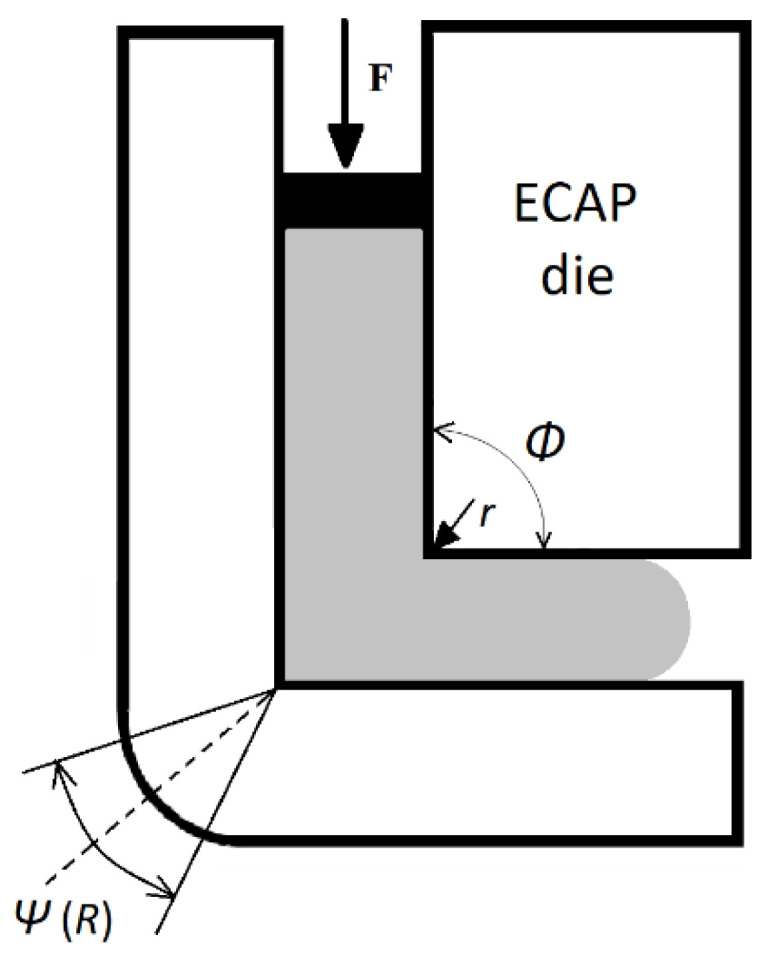
Diagram of the ECAP die used [28].

A simplified drawing of the die and its angles is shown in Figure 2. The experiments were performed with a pressing speed of 1.73 mm·s^−1^.

The aim of these experiments was to determine the strength of the material after each pass, as well as the dependence of the change in structure (grain size) and mechanical properties (tensile strength, yield point, Young’s modulus, etc.).

For the measurement of forces in the ECAP process, a strain gauge resistive sensor type 3/350 XK 11 designed at VŠB Ostrava (Czech Republic) was used, which measures forces up to 1000 kN with an accuracy of 0.5%. Its nominal resistance is R1–R8 = 350 Ω. This sensor is one of the foil strain gauges used for precise measurements of deformations, pressures, moments, and forces acting on the tested bodies. Its principle is to detect the deformation of a mechanically stressed conductor (in this case, the extruded sample) that causes a change in geometric dimensions. This results in a linearly varying resistance, the progression and conversion of which into measured information is provided by other devices.

**Figure 3:**
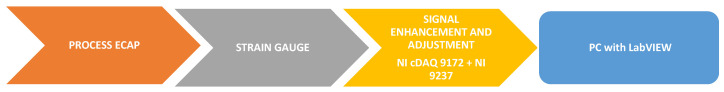
Block diagram of force measurement in the case of the ECAP technology.

The NicDAQ laboratory instrument and LabView software from National Instruments were used to amplify and modify the strain gauge signals. The samples were processed sequentially through four passes of the ECAP die. Figure 3 shows the flow of information from the experiment up to the level of processing in the PC program.

### 2.3. Metallographic Analysis

In order to increase the visibility of the microstructure (morphology) of the material, the samples were ground on a KOMPAKT 1031 grinder with 600-, 1200-, and 2000-grit MESH sandpaper. The term “MESH” is a grain size expressed as a number that indicates the number of meshes per inch (25.4 mm) that the abrasive will still pass through during sieving. Thus, the higher the grain size number, the denser the sieve and the finer the abrasive. The device was switched on when the water supply began, and its rotation speed (rpm) could be continuously varied by a potentiometer. The sample was successively sanded with sandpaper, in the order of coarsest to finest, rinsing it each time with water and ethanol to remove contaminants effectively.

The samples were then polished for 20 s in a Lector Pol-5 device. This is an electrolytic polisher manufactured by Struers that allows both electrolytic polishing and etching. The polishing was carried out under a DC current of 24 V in an electrolyte referred to by the company as D2.

Electrolytic polishing is not as versatile as mechanical polishing. Therefore, it is used as in the presented case, especially for soft materials, which can easily form grooves and a thick Beilby layer during conventional grinding. This continuous layer (also referred to as the B-layer) represents the strain-hardening material on the surface of the sample that forms when it is mechanically impacted. Finally, the samples were chemically etched for 4 s in the same Struers LectroPol-5 device under a DC current of 2 V in electrolyte D2. The etching process enhances the surface structures due to the disruption of the grain boundaries, allowing a thorough investigation of the grain size. However, the method used does not result in colour differentiation of individual grains. An inverted metallographic microscope GX51 was used for visual observation of the metallographic grinding and polishing. This instrument allows examination of the material by means of the reflected light in the bright field, reflected light in the dark field, reflected light in the interference differential contrast, and reflected polarised light.

The samples were observed under reflected light with a maximum magnification of 1000×. In this method of examining a sample, the surface of the material is illuminated and then (similar to reflection in a mirror) the light reflected from its surface can be observed.

### 2.4. Measurement of Mechanical Properties

**Figure 4:**
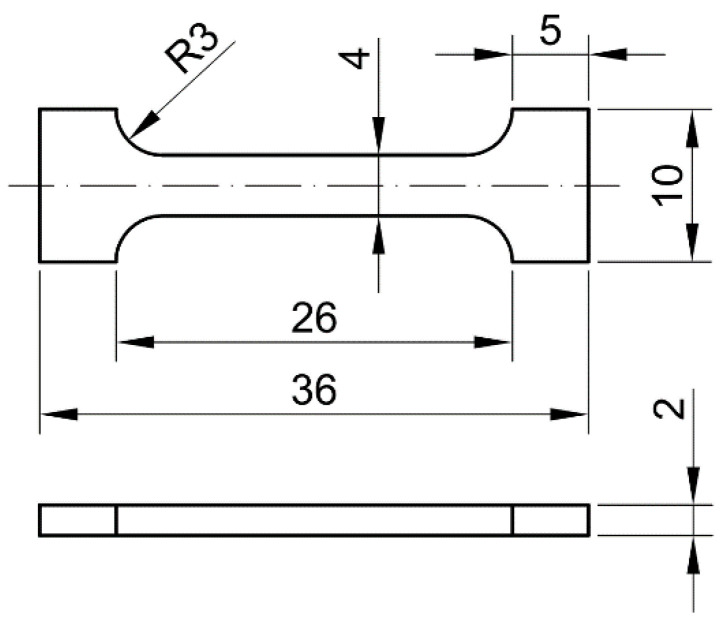
Drawing of the test bar used in tensile tests (dimensions are in millimeter).

In order to perform tensile tests on the samples, modified bars of non-normalised samples with rectangular cross-sections were constructed after being pushed through the die. The tests were carried out in a specialised accredited company, namely in the testing laboratory of VÚHŽ a.s. Dobrá (Czech Republic). This laboratory has developed a methodology for tensile testing on non-standard bars with verification of the repeatability of the results. Figure 4 shows the test samples used for the tensile test and their dimensions. For each group of samples evaluated, 2 test pieces were produced.

The applicable normative procedures were followed as much as possible when performing the tensile test to determine the required parameters. Several necessary modifications to the test methodology were made within the permitted standards due to the limited dimensions of the non-standard test pieces used.

## 3. Results

### 3.1. Yield Point, Breaking Stress, and Ductility

Methodologically, the determination of the yield point $R_{p0.2}$, breaking stress Rm, and ductility *A* was carried out by modifying the standard EN 10002-1. Due to the size of the sample, no extensometer was used (strain sensing was realised by electronic transverse scanning at 250× magnification with an accuracy of ±0.5 µm). The calculations of the mechanical values were carried out in accordance with EN ISO 6892-1:2009.

### 3.2. Young’s Modulus of Elasticity

Young’s modulus *E_mat_* was determined in accordance with the standard ASTM E-111 [29]. This test method makes it possible to determine not only Young’s modulus but also other important quantities of structural materials.

### 3.3. Determination of the Poisson’s Ratio µ

In the tests, the Poisson ratio was determined by measuring the deformation in the transverse and longitudinal direction in the proportional deformation region and then processing the measured deformation waveforms. Its values, together with the other quantities, are given in Table 2.

Due to the small size of the test bodies, it was not possible to use the extensometer in either of the two mutually perpendicular directions. Therefore, the longitudinal strain was measured in the same way as for ductility, yield point, and strength, i.e., by electronic sensing from the crosspiece at 250× magnification and with an accuracy of ±0.5 µm).

### 3.4. Discussion of Test Results

**Figure 5:**
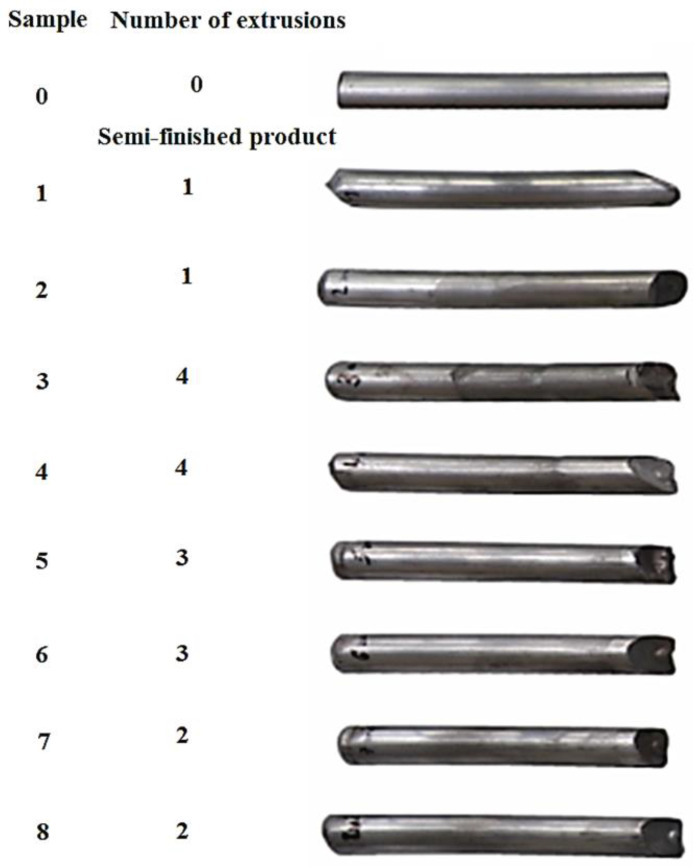
Sample set prepared by the ECAP method.

Figure 5 shows a set of samples generated by the ECAP method. A total of four pairs of samples were extruded and subjected to the first to fourth extrusion. After processing a total of 20 passes through the ECAP process, tests were performed on each pair of samples to determine their properties. The first sample from each pair was used for metallographic examinations and the second for the determination of mechanical properties.

### 3.5. Measurement of Forces

The following graphs show the dependence of *F* = f(*L*) for the EN AW 6060 alloy when extruded through the die. Since all eight tested samples had identical dimensions at the beginning of the experiments, the change in length can be very well observed from the graphs. The extrusion forces were measured sequentially depending on the extruded length of the samples. Two samples were always compared after each pass. During each extrusion, the edges of the samples were deformed significantly.

**Figure 6:**
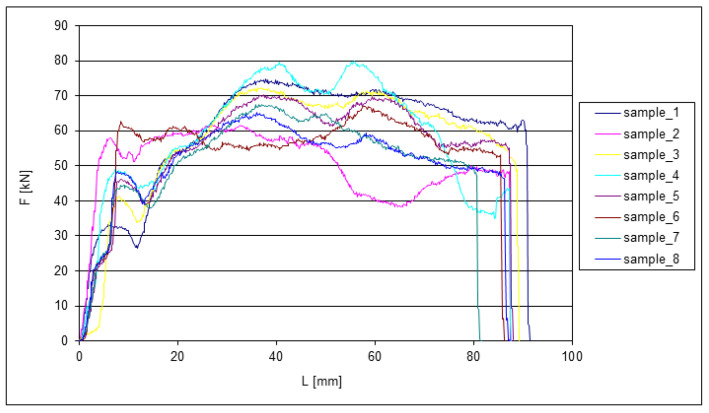
Dependence of the force on the length of the extrusion during the first pass through the ECAP die.

It can be observed that the curves in Figure 6 have a large dispersion of deformation forces at the first pass of the ECAP. The dispersion of forces *F_max_* was up to 23 kN, which represents 28% of the measured range.

**Figure 7:**
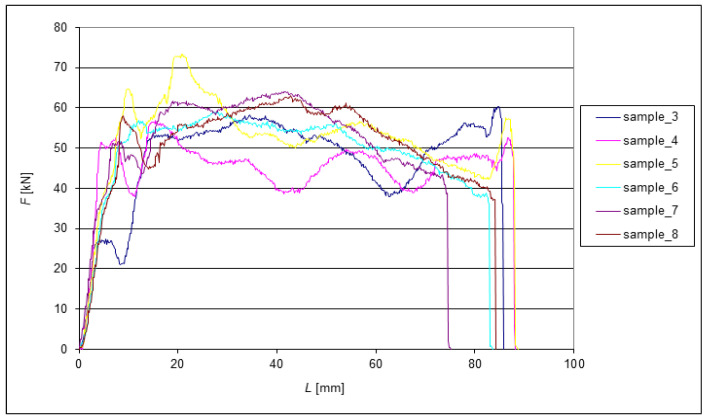
Dependence of the force on the length of extrusion during the second pass through the ECAP die.

**Figure 8:**
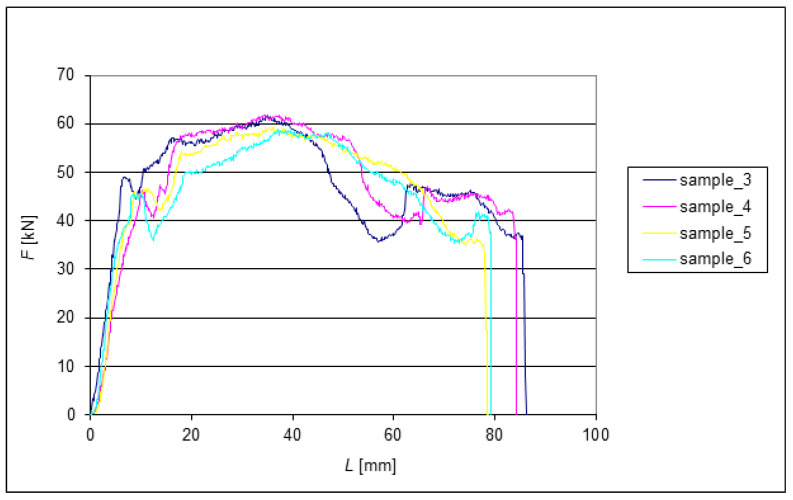
Dependence of the force on the length of extrusion during the third pass through the ECAP die.

Figure 7 shows the subsequent reduction in dispersion *F_max_* to 12.5% of the measured force range after the second extrusion. As expected, there was a general, albeit small, reduction in the sample length. Compared to the other samples, greater shortening was observed for Sample 7 after only two extrusions. To some extent, this may have been due to measurement inaccuracy due to irreducible deformation of the end of the samples. Figure 8 shows the force waveform after the third ECAP pass.

**Figure 9:**
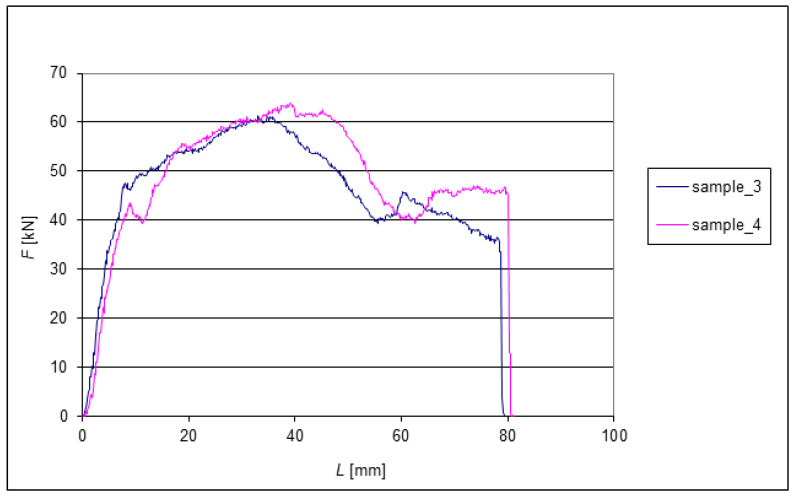
Dependence of the force on the length of extrusion during the fourth pass through the ECAP die.

Figure 9 shows the forces during the final extrusion of the last pair of Samples 3 and 4. However, there was no significant dispersion of forces $F_{max}$. Their dispersion is 2.5 kN, which is approximately 4%. The final length of the two samples differed by approximately 1.5 mm.

After completing the specified number of extrusions of all samples by the ECAP method and recording the values with a strain gauge, metallographic research and mechanical property tests followed. The behaviour of the compressed material is aptly described by the Hall–Petch relationship, which can be generalised to various materials, including the material under study. There is a grain size limit within which the strength stops increasing or even begins decreasing. Within this limit, the structure of the materials is fragmented, so a further increase in stress is undesirable.

### 3.6. Results from Metallographic Tests

**Figure 10:**
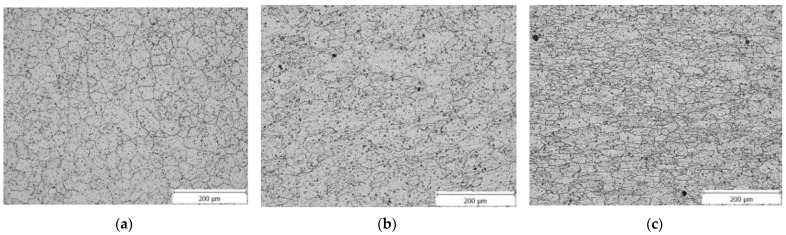
(**a**) Microstructure of the initial material (Sample 0); (**b**) microstructure of the material after one pass through the ECAP die (Sample 1); (**c**) microstructure of the material after four passes through the ECAP die (Sample 4).

Measurements of medium grain size were made in two directions. Figure 10a shows the microstructure of the initial material. It shows that the microstructure is homogeneous and without obvious anisotropy. The mean grain size is relatively large for the undeformed samples, approximately 28.9 μm. The microstructure of the material after one pass through the ECAP die can be seen in Figure 10b. The mean grain size of the sample is 12.51 μm after one pass. Figure 10c shows the microstructure of the material after four passes through the ECAP die; the mean grain size is 4.63 µm.

The intense plastic deformation during extrusion obviously has a great impact on the microstructure and grain size of the processed materials. In addition to the temperature and strain rate, the magnitude of the stacking fault energy (SFE) has an impact on the formation of the resulting microstructure during the SPD process and the movement of dislocations through the material. Stacking faults are among the surface defects in f.c.c. lattice metals that can commonly occur during forming. The relatively large SFE, which is typical for aluminium alloys, prevents complete dislocation during deformation, and only partial (incomplete) dislocation occurs. Moreover, even the dislocation slip, in which dislocations move in slip planes in the material, is limited due to cold forming.

### 3.7. Mechanical Properties

Table 2 shows the values of mechanical properties determined from tensile tests. These are the average values of the mechanical quantities determined from two tests for each individual group of samples.

**Figure 11:**
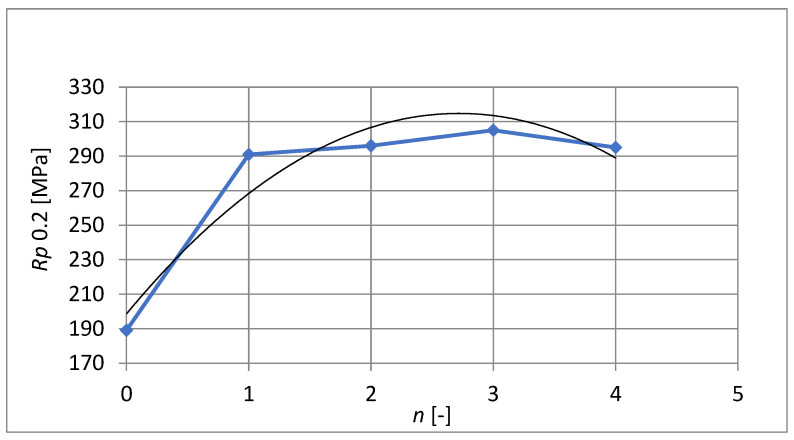
Yield point after individual extrusions.

Figure 11 shows the yield point course versus the number of passes through the ECAP die. The greatest increase in the yield point is achieved after the first pass through the ECAP die, where the value increases from *R_p*0.2*_* =189 MPa to *R_p*0.2*_* = 291 MPa, an increase of more than 50%. Subsequent extrusions led to a minimal increase in the yield point and then a decrease after the fourth extrusion.

In addition to the yield point value itself, Figure 11 also shows a polynomial regression curve determined by the least-squares method. With this dependence, the parameter of the largest coefficient of determination is determined as *R*^2^ = 0. 9118(5):

(5) $$Rp_{0.2}=-15.714\cdot n^{2}+85.457\cdot n+198.57$$

**Figure 12:**
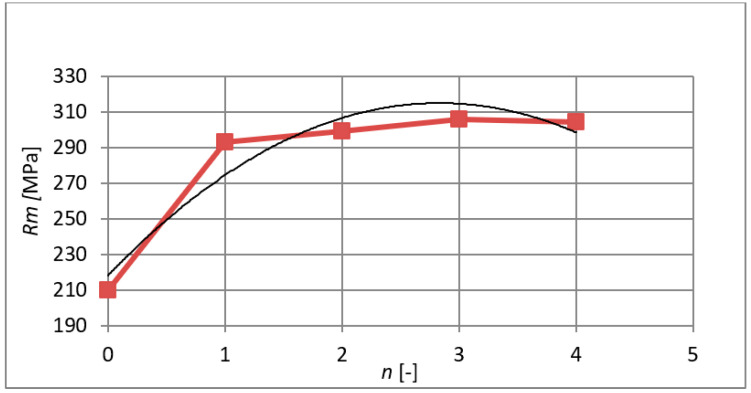
The course of the ultimate tensile strength after individual extrusions.

Figure 12 shows the course of the ultimate tensile strength and its gradual increase after each extrusion. The maximum coefficient of determination in this case is *R*^2^ = 0.9139, and the calculation of the regression curve is (6):

(6) $$Rm=-12.071\cdot n^{2}+68.386\cdot n+218.060$$

**Figure 13:**
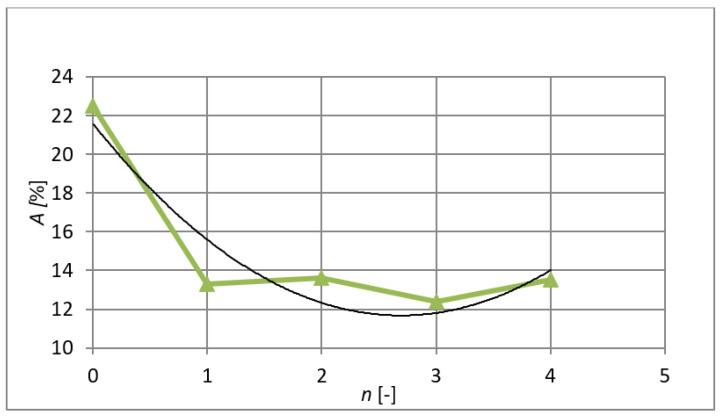
The course of ductility after individual extrusions.

In addition to a number of improved properties, fine-grained and nanocrystalline materials are often characterised by reduced ductility [20]. However, this is also visible in Figure 13, where it can be seen that in contrast to the increasing breaking stress and yield point after extrusions by the ECAP method, the ductility of the material is significantly reduced by almost 10%. It is not possible to state unequivocally whether this is due to poor consolidation or whether some intrinsic physical reason is responsible. The largest coefficient of determination is *R*^2^ = 0.8814. The regression curve in this case is (7):

(7) $$A=1.364\cdot n^{2}-7.347\cdot n+21.569$$

**Figure 14:**
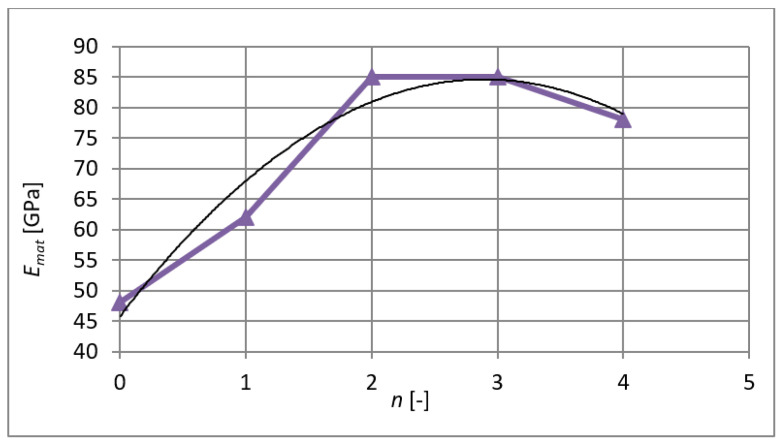
Evolution of the modulus of elasticity after individual extrusions.

The modulus of elasticity during extrusions is shown in Figure 14 with the regression curve, whose largest coefficient of determination in this case is *R*^2^ = 0.9442. It can be seen that together with the values of yield stress and strength at the last pass through the die, there is also a slight decrease in the elasticity values. The polynomial regression curve is (8):

(8) $$E_{mat}=-4.643\cdot n^{2}-26.871\cdot n+45.714$$

The quantity R expresses the tightness of the agreement according to the least-squares method to demonstrate extrusion trends, i.e., analytically (using a spreadsheet editor), the expression of the interleaved function and the objective, reproducible determination of the best match criterion is visible. The given regression equations were generated to determine the extrusion trend of a particular stressed material, not to predict material parameters in general.

## 4. The Interpretation and Solution Method from the Obtained Results

The notion that pushing a sample through a fractured channel induces an intense plastic deformation *ε_PLn_* in the material is an input for the solution. In the knee of the channel, the yield point *Re_n_* ≥ *Re_o_* is exceeded quite suddenly, and the layers of structural grains *D_gr_* are subsequently displaced relative to each other with shape transformations of their original volume. The magnitude or intensity of the volume and shape deformations primarily depend on a number of key variables, according to the implicit Equation (9):

(9) $$D_{grecn}=(D_{gro},D_{grn},Re_{o},Re_{n},E_{mo},n,E_{mn},F_{ecn},\varepsilon_{PLn},\varepsilon_{PLno},\varepsilon_{PLecn},\varepsilon_{nre},S_{ecj},V_{ec},V_{def},n_{o}\ldots )$$

The parameters *D_gro_*, *R_eo_*, and *E_mo_* represent the material values in the unloaded state.

The symbols used in Equation (9):
*D_gro_*, *D_grn_*original and transformed structural grain diameter, (µm);*Re_o_*, *Re_n_*original and transformed yield point (MPa);*E_mo_*, *E_mn_*original and transformed Young’s modulus (MPa);*F_ecjn_*needed unit extrusion force per sample area *S_ecj_* = 1 mm^2^, (N);*F_ecn_*needed extrusion force per sample area *S_ec_* in mm^2^, (N);*ε_nre_*relative plastic deformation of the structural grains at the level of *R_en_* (-);*ε_PLno_*, *ε_PLn_*original and transformed relative plastic deformation of structural grains, positive branch, (-);*ε_PLecn_*relative plastic deformation of structural grains, negative branch, (-);*V_ec_*speed of pushing through the channel, (m⋅s^−1^);*V_def_*strain rate of the sample material, (s^−1^);*n*required number of extrusions for the desired *D_grn_*/*D_gro_* ratio, (-);*n_o_*number of pushes on the neutral plane (-).

The set of declared variables (9) can be further interactively expanded according to the actual problem of interest, e.g., according to other related structural material, thermodynamic, crystallographic, and other constraints. Although this is a relatively simple measurement principle, its laboratory implementation can provide a wide range of technically important properties for the practical use of a particular material.

### Derivation of Mathematical Functions for the Analytical Solution of a Process

The challenge is to make explicit the parameters given by the implicit Equation (9). The analytical solution of the extrusion process of material samples by the ECAP method is based on the newly derived equations [30,31,32] of equilibrium state ratios (10)–(12).

Explicit connections between the most important process parameters reported in (9):

(10) $$\frac{F_{ecjn}}{Re_{o}\cdot S_{ecj}}=\frac{\varepsilon_{PLn}}{n}$$

(11) $$\frac{\varepsilon_{PLn}}{n}=\frac{D_{grn}}{D_{gro}}$$

and, therefore,

(12) FecjnReo⋅Secj=εPLnn=DgrnDgro

It follows that the most important process parameters can be considered to be the parameters included in the partial equations of equilibrium (10)–(12), and their mutual physical–mechanical connections, including the partial connections. These linkages physically and mathematically imply the occurrence of both increasing and decreasing equations. In principle, the decreasing function for the structural grain size *D_grecn_* (18) is fundamental. The process reduction in the grain diameter is adequately matched by the growth of the strength, elasticity, and hardness functions of the material over the various cycles of the sample extrusion through the channel. The terms (1 + *ε_nre_*) and 1/(1 + *ε_nre_*) can be called the growth or decrease factors of the functions in the process. The individual parameters can be obtained in exact forms by isolation. For the required unit extrusion force per sample area, relation (13) holds:

(13) $$F_{ecjn}=\frac{D_{grn}}{D_{gro}}\cdot Re_{o}\cdot S_{ecj}$$

The required extrusion force [32] per the sample area is given by (14) and (15):

(14) $$F_{ecn}=\frac{D_{grn}}{D_{grno}}\cdot Re_{o}\cdot S_{ec}$$

(15) $$F_{ecn}=F_{eco}\cdot \left(1+\varepsilon_{nre}\right)$$

The transformed diameter of the structural grain can be determined according to relations (16) and (17):

(16) $$D_{grn}=\frac{F_{ecn}\cdot D_{grno}}{Re_{o}\cdot S_{ec}}$$

(17) $$D_{grn}=D_{gro}\cdot \left(1+\varepsilon_{nre}\right)$$

The transformed diameter of the structural grain can be determined according to relation (18):

(18) $$D_{grecn}=\frac{D_{gro}}{\left(1+\varepsilon_{nre}\right)}$$

For the transformed yield point, Equation (19) applies:

(19) $$Re_{n}=Re_{o}\cdot \left(1+\varepsilon_{nre}\right)$$

The relative plastic deformation of the structural grains (positive branch) is given by Equations (20) and (21):

(20) $$\varepsilon_{PLn}=\frac{F_{ecjn}}{Re_{o}\cdot n}\cdot S_{ecj}$$

(21) $$\varepsilon_{PLn}=\frac{D_{grn}}{D_{grno}\cdot n}$$

The relative plastic deformation of the structural grains (negative branch) is given by (22):

(22) $$\varepsilon_{PLecn}=\varepsilon_{{}_{PLn}}^{-1}$$

The transformed Young’s modulus can be determined according to Equation (23):

(23) $$E_{mn}=E_{mo}\cdot \left(1+\varepsilon_{nre}\right)$$

The speed of pushing through the channel is determined by Equation (24):

(24) $$V_{ec}={\left(10^{-3}\cdot Ra_{o}\right)}^{0.5}\cdot \frac{10^{6}}{E_{mat}{}^{0.5}}$$

The deformation rate of the sample material can be determined according to Equation (25):

(25) $$V_{def}=\frac{\varepsilon_{PLn}}{t}$$

where *t* is the deformation time (s).

The required number of extrusions *n* (-) can, therefore, be determined in advance by calculation according to relations based, for example, on the proportionality Equations (10) and (11), from which the Equations (26) and (27) follow:

(26) $$n=\frac{Re_{o}\cdot D_{grn}}{D_{gro}\cdot F_{ecn}}\cdot S_{ecj}$$

(27) $$n=\frac{Re_{o}\cdot \varepsilon_{PLec}}{F_{ecn}}\cdot S_{ecj}$$

The number of pushes on the neutral plane can be determined from relation (28):

(28) $$n_{o}=\frac{\varepsilon_{PLn}}{D_{grn}}\cdot D_{grno}$$

The relations for *n* are, therefore, derived in an exact way and can be conveniently used to check the parameter calculations performed in the extrusion project (Table 3).

**Figure 15:**
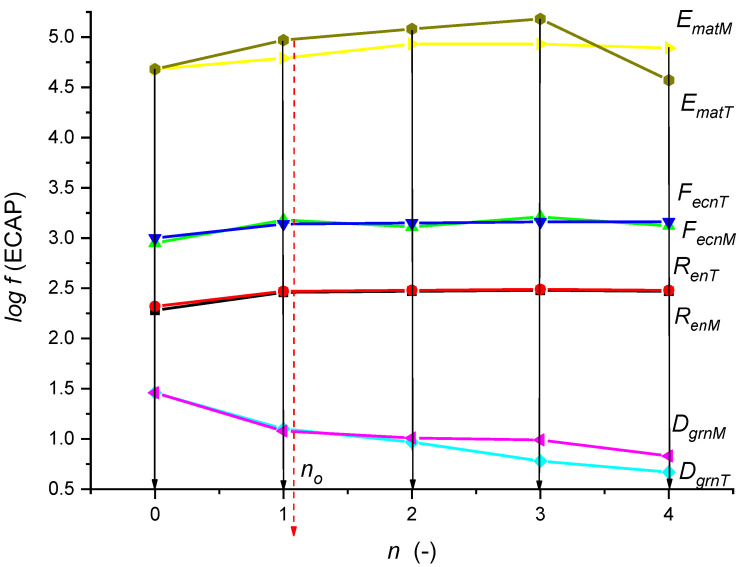
Comparison of measured and theoretical values for *n* = 1 to *n* = 4 extrusions (Table 3).

A graphical comparison of the measured and theoretical values was made in decadic logarithms (Figure 15). A very good consensus of the compared data can be seen.

According to the graph in Figure 15, for all the compared functions, there is a minimum difference between the measured and theoretical values. This fact justifies accepting the proposed and newly developed theory and offering it for use in laboratory and technical practice.

In the used experimental ECAP device, the so-called backpressure, which did not act on the opposite surface of the sample outlet end, was not used against the extrusion pressure (working on the area of the sample inlet end). Without the effect of backpressure, the ends of the samples are always significantly deformed (especially when the outer bend of the extrusion elbow is shorter than the inner bend, see Figure 2).

From the principle of material mechanics, it is, in our opinion, an inevitable effect. The reason is due to:

(a) External influence from the formation of crushing on the surfaces of direct contact with the extruder and the channel walls at extreme pressure in the knee.
(b) Internal influence from alterations of the state of the mechanical structure of the sample material as it passes through the knee under extreme pressure.
(c) Due to a significant number of related variables (see Equation (9) and others), any objective or subjective errors cannot be completely excluded for the measured parameters.

**Figure 16:**
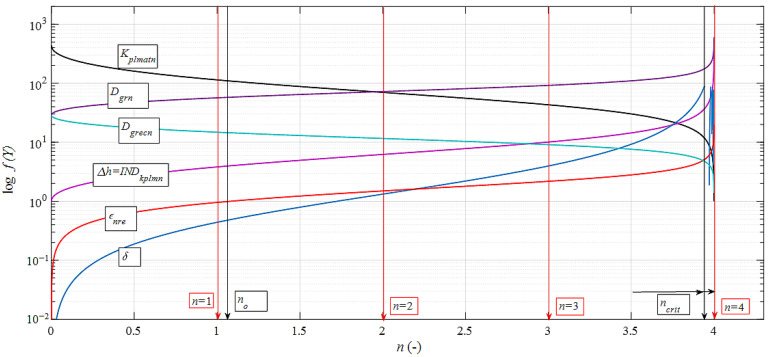
Logarithmic dependence of prediction functions on the number of extrusions.

In particular, the influence of internal alterations ad b) is complex in material mechanics and depends on several variables. In this paper, we tried to manage it analytically in a form that can be used both in theory and technical practice. Our own newly derived equation for reducing samples Δ*h* is confirmed in our application works. It describes the general fact that the brittleness increases with increasing strength, and thus, the deformation is shortened (29):

(29) $$K_{plmatn}=\frac{10^{12}}{{\left(E_{mat}\cdot \left(1+\varepsilon_{nre}\right)\right)}^{2}}$$

where the plasticity index is (30):

(30) $$IND_{kplmn}=\frac{K_{plmat}}{K_{plmatn}}$$

and the shortening in an absolute value is Δ*h* = *IND_kplmn_*. The relative deformation *ε_nre_* (-) is also given by the measurable ratio *D_gro_*/*D_grn_* (-). An example is the immediate alteration of *D_grn_* as it passes through the elbow of the extrusion channel. According to our theoretical ideas, the solution principle is illustrated by the graph in Figure 16.

**Figure 17:**
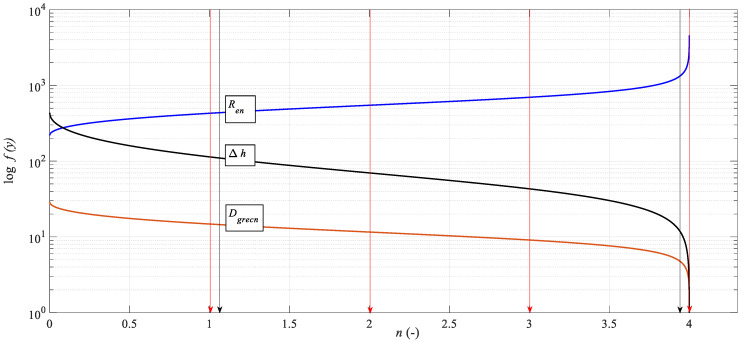
Dependence of grain reduction and other functions on the degree of extrusion.

The microstructure of the alloy under investigation consists of a significant number of grains separated by small angular grain boundaries. These grains are stretched into a band structure. Subsequent extrusion increases the applied stress, causes a reduction in grain size, and weakens the band structure. The degree of breakage of the band structure also depends on how the ECAP process is performed. For the selected process, the band structure remains until the fourth pass through the matrix, i.e., there is a significant refinement of the structure, mainly due to shear between the grains. Yield point *R_p*0.2*_* [MPa] increased by 56%, ultimate tensile strength *Rm* [MPa] increased by 45%, ductility *A* [%] decreased to 60%. Grain reduction is limited by reaching the limit angle of internal friction *δ* = 90° (see Figure 17) and thus at the level of critical *n_crit_*. The yield strength increases, and at the same time, the material becomes brittle and its ductility decreases. It can be seen from the graph that where the strength *R_en_* = f(*n*) increases adequately according to the decreasing diameter *D_grn_* = f(*n*), the deformation length of the material *K_plmatn_* = f(*n*) also decreases accordingly. A decrease in grain size is not indicative of corresponding changes in material parameters because grain reduction is limited; extreme grain fragmentation would be undesirable and would not lead to improved material properties.

## 5. Verification by Hall–Petch Relation

**Figure 18:**
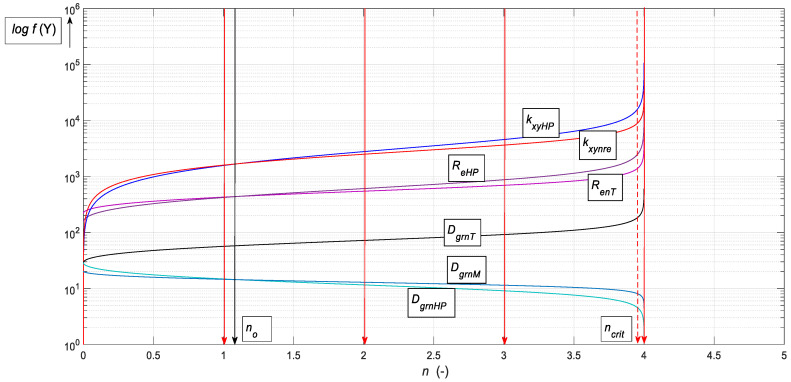
Verification comparison for the specific case of our experiment and newly presented theories according to the Hall–Petch relation.

Due to the importance and continuing widespread use in engineering practice, a detailed verification comparison was made to Equation (1) for the specific case of the presented experiment and the presented theory according to the Hall–Petch relation. It is graphically represented by the graph in Figure 18.

On the neutral plane, the material constant *k_xy_* for the value determined by *Re* is equal to the material constant according to the Hall–Petch relation *k_xynreo_* = *k_xyhpno_ =* 1.672, while the yield point according to Hall–Petch is equal to the yield point according to the relation determined by *R_eHPo_* = *R_enT_* = 219 MPa; thus, for the relative plastic deformation of the structural grains on the neutral level *R_en_, ε_nreo_* = 1 applies.

For the material constant on the neutral plane, Equation (31) applies:

(31) $$k_{xynreo}=k_{xyo}\cdot \varepsilon_{nreo}$$

For the transformed material constant, Equations (32) and (33) apply:

(32) $$k_{xynre}=k_{xynreo}\cdot \varepsilon_{nre}$$

(33) $$k_{xynre}=k_{xyo}\cdot \varepsilon_{nre}$$

For the transformed material constant, which reflects the relative plastic deformation, Equations (34) and (35) apply:

(34) $$k_{xyHPn}=k_{xyHPno}\cdot \left(1+\varepsilon_{nre}\right)$$

(35) $$k_{xyHP}=D_{grn}^{0.5}\cdot \left(R_{en}-R_{eHPo}\right)\cdot 2$$

As shown in the graph in Figure 18, systematic differentiation results for all the functions are compared. It can be seen that identical values are obtained at the level of the neutral parameter *n_o_*. This systematic difference is related to the opinions of a number of experts who point out a certain inaccuracy of the calculations according to (1) in regions of a very small structural grain size compared to larger grain size.

One of the critics of the Hall–Petch relation (1) is Meshal Y. Alawadhi [26]. Therefore, a comparison of the presented results according to [26] in parameter *k_xy_* from Equation (1) and also according to *k_xynre_* by the authors’ equation (33) was also made. In the original [20], one can also find support in the laboratory measurements in the plots in Figure 8 and Figure 9. All the results presented are correlated.

## 6. Conclusions

The aim of the presented publication was to investigate the structural and mechanical changes of the selected material, namely aluminium alloy EN AW 6060, after processing by the ECAP method. Based on the experimental results obtained, it is then possible to describe the physical–mechanical principle of the process for the exact design, measurement control, and strengthening of the material for applications in engineering practice analytically.

Due to its relative ductility at room temperature, the investigated aluminium alloy is ideal for channel extrusion using the ECAP method, namely through a rectangular extrusion channel. It has been confirmed that the chosen extrusion route C, where the sample is rotated 180° after each pass, can create a homogeneous fine-grained structure in the material and provide relatively uniform deformation throughout the sample volume. After metallographic investigation, changes in the grain size and shape were observed. The mean grain size decreased from a value of 28.9 µm in the original material to 4.63 µm after the fourth ECAP pass, with a corresponding decrease in ductility from 22.5% to 13.6% and an increase in strength from 210 MPa to 304 MPa.

This refinement of the microstructure after processing by the ECAP method is the reason for the changes in mechanical properties. After the first extrusion, it has been shown that there is already a significant improvement in strength and toughness and a significant reduction in ductility. After further extrusions, these values changed to only a small extent until the fourth extrusion, when the strength and flexibility of the material decreased slightly due to the appearance of micro-cracks.

By investigating the relationship between the development of a structural and mechanical material change during the extrusion of the AlMgSi_0.5_ alloy by ECAP with respect to the use of knowledge in the design phase, extrusion technology can be designed and controlled more effectively to achieve the desired grain size and material hardening based on the newly acquired knowledge.

In summary, the following results were achieved:

- Regression equations were developed based on the tensile test measurements (5)–(8);
- On the basis of the explicit Equation (9), mathematical functions for the analytical solution of the process were successively derived in order to predict (10)–(30);
- Graphical comparisons of measured and theoretical values in decadic logarithms were performed (Figure 15), showing an excellent consensus of the compared data, namely within 5%;
- Verification comparison with the Hall–Petch relation was performed, from which new prediction Equations (31)–(35) were obtained.

Nanostructured metallic materials undoubtedly lead to new directions in the fabrication of complex-shaped micro components for microdevices. Aluminium alloys with a more ultrafine microstructure show enhanced static and cyclic strength, a higher strength-to-weight ratio, as well as more extensive application and multifunctional properties. For these reasons, they are increasingly in demand as structural and functional materials in various engineering sectors. Extensive current and future use areas continue to include aerospace, construction, automotive, military, and electronics.

## Figures and Tables

**Table 1:** Chemical composition of aluminium alloy AlMgSi_0.5_ (EN AW 6060).

Material %		Si	Fe	Cu	Mn	Mg	Cr	Zn	Ti	Al
AlMgSi_0.5_	min	0.3	0.1			0.35				Rest.
	max	0.6	0.3	0.10	0.10	0.60	0.05	0.15	0.10	

**Table 2:** Results of mechanical properties.

Number of Extrusions	Yield Point *R_p*0.2*_* [MPa]	Ultimate Tensile Strength *Rm* [MPa]	Ductility *A* [%]	Modulus of Elasticity *E_mat_* [GPa]	Poisson’s Ratio *µ* [-]
0	189	210	22.5	48	0.4
1	291	293	13.3	62	0.35
2	296	299	13.5	85	0.30
3	305	306	12.4	85	0.30
4	295	304	13.6	78	0.30

**Table 3:** Comparison of measured and theoretical values.

*n*	*R_enM_*	*R_enT_*	*F_ecnM_*	*F_ecnT_*	*D_grnM_*	*D_grnT_*	*E_matM_*	*E_matT_*
0	189	219	888	989	28.90	28.90	48,000	48,000
1	291	293	1509	1380	12.51	11.90	62,000	93,910
2	296	299	1296	1408	9.23	10.30	85,000	11,9800
3	305	306	1630	1441	5.97	9.80	85,000	152,600
4	295	304	1316	1432	4.63	6.70	78,000	36,980
log *f*								
***n***	***R_enM_***	***R_enT_***	***F_ecnM_***	***F_ecnT_***	***D_grnM_***	***D_grnT_***	***E_matM_***	***E_matT_***
0	2.28	2.32	2.95	3.00	1.46	1.46	4.68	4.68
1	2.46	2.47	3.18	3.14	1.10	1.08	4.79	4.97
2	2.47	2.48	3.11	3.15	0.97	1.01	4.93	5.08
3	2.48	2.49	3.21	3.16	0.78	0.89	4.93	5.18
4	2.47	2.48	3.12	3.16	0.67	0.72	4.89	4.57

## Data Availability

The data presented in this study are available on request from the corresponding author.

## Nomenclature

**Symbol**		**Measurement Unit**
*σ*	total stress in the material	MPa
*σ_y_*	yield strength	MPa
*σ_o_*	frictional stresses required for the movement of dislocations,	MPa
*σ_GB_*	grain boundary reinforcement	MPa
*σ_dis_*	dislocation hardening	MPa
*k_xy_*	material constant	N∙mm^−3/2^
*k_xyHP_*	transformed material constant	N∙mm^−3/2^
*k_xy HPn_*	transformed material constant after *n* extrusions	N∙mm^−3/2^
*k_xy HPno_*	transformed material constant at neutral level	N∙mm^−3/2^
*d*	mean grain diameter	mm
*D_gro_*	original diameter of the structural grain in the unloaded state,	μm
*D_grn_*	transformed diameter of the structural grain after *n* extrusions	μm
*D_grecn_*	transformed diameter of the structural grain	μm
*α*	numerical factor depending on the dislocation arrangement	-
*M*	Taylor factor	-
*G*	modulus of elasticity in shear	Pa
*b*	Hamburger vector for edge dislocation	mm^−1^
*ρ*	dislocation density	m^−2^
*Φ*	angle of the channel with internal curvature *r*	
*Ψ(R)*	angle of external curvature *R* of the channel	
*L*	sample length	mm
*n*	number of extrusions	-
*n_o_*	number of extrusions on the neutral plane	-
*IND_kplmn_*	index plasticity	-
*A*	ductility	%
*t*	time	s
*µ*	Poisson number	-
*Rm*	ultimate tensile strength	MPa
*Re_o_*	original yield point in unloaded condition	MPa
*Re_n_*	transformed yield point	MPa
*R_eHPo_* = *R**_enT_*	yield point according to the Hall-Petch relation	MPa
*F*	extrusion force	kN
*F_ecjn_*	required unit extrusion force per sample area *S_ecj_* in mm^2^	kN
*F_ecn_*	necessary extrusion force per sample area *S_ec_* in mm^2^	kN
*F_max_*	dispersion of forces	kN
*E_mat_*	Young’s modulus of elasticity	MPa
*E_mn_*	transformed Young‘s modulus of elasticity after *n* extrusions	MPa
*E_mo_*	original Young‘s modulus of elasticity in the unloaded state	MPa
*ε_nre_*	relative plastic deformation of structural grains at the level of *R_en_*	-
*ε_PLn_*	relative plastic deformation of structural grains, positive branch	-
*ε_PLec_*	relative plastic deformation of structural grains, negative branch	-
*ε_nreo_*	relative plastic deformation of structural grains at neutral level	-
*V_ec_*	speed of extruding through the channel	mm·s^−1^
*V_def_*	rate of the sample material deformation	s^−1^

## References

[1] Langdon T.G., Furukawa M., Nemoto M., Horita Z. (2000). Using equal-channel angular pressing for refining grain size. JOM.

[2] Gupta A., Chandrasekhar B., Saxena K.K. (2021). Effect of Equal-channel angular pressing on mechanical properties: An overview. Mater. Today Proc..

[3] Zhang Q., Li Q., Chen X. (2021). Research progress of ultrafine grained magnesium alloy prepared by equal channel angular pressing. Mater. Res. Express.

[4] Ka U.B., Panemangalorea D.B., Bhatb S., Davim J.P., Gupta K. (2021). Equal channel angular processing—A modern deforming technique for quality products. Advanced Welding and Deforming.

[5] Snopiński P., Woźniak A., Pagáč M. (2021). Microstructural Evolution, Hardness, and Strengthening Mechanisms in SLM AlSi10Mg Alloy Subjected to Equal-Channel Angular Pressing (ECAP). Materials.

[6] Frint S., Hockauf M., Frint P., Wagner M.F.X. (2016). Scaling up Segal’s principle of equal-channel angular pressing. Mater. Des..

[7] Zehetbauer M., Valiev Z.A. (2004). Nanomaterials by Severe Plastic Deformation.

[8] Janeček M., Krajňák T., Stráská J., Čížek J., Lee D.J., Kim H.S., Gubicza J. (2014). Microstructure evolution in ultrafine-grained interstitial free steel processed by high pressure torsion. IOP Conf. Ser. Mater. Sci. Eng..

[9] Sanusi K.O., Makinde O.D., Oliver G.J. (2012). Equal channel angular pressing technique for the formation of ultra-fine-grained structures. S. Afr. J. Sci..

[10] Altan B. (2006). Severe Plastic Deformation: Toward Bulk Production of Nanostructured Materials.

[11] Nashith A., Sanjid P., Shamsudheen M., Rasheeque R., Ramis M.K., Shebeer A.R. (2014). Effect of equal channel angular pressing (ECAP) on hardness and microstructure of pure aluminum. Int. J. Mater. Eng..

[12] Valiev R.Z., Islamgaliev R.K., Alexandrov I.V. (2000). Bulk nanostructured materials from severe plastic deformation. Prog. Mater. Sci..

[13] Huang Y., Langdon T.G. (2013). Advances in ultrafine-grained materials. Mater. Today.

[14] Ilucová L., Saxl I., Svoboda M., Sklenička V., Král P. (2007). Structure of ECAP aluminium after different number of passes. Image Anal. Stereol..

[15] Gopi K.R., Shivananda Nayaka H., Sahu S. (2017). Wear properties of ECAP-processed AM80 magnesium alloy. J. Mater. Eng. Perform.

[16] Figueiredo R.B., Cetlin P.R., Langdon T.G. (2007). The processing of difficult-to-work alloys by ECAP with an emphasis on magnesium alloys. Acta Mater..

[17] Nakashima K., Horita Z., Nemoto M., Langdon T.G. (1998). Influence of channel angle on the development of ultrafine grains in equal-channel angular pressing. Acta Mater..

[18] Jha S.K., Balakumar D., Paluchamy R. (2015). Experimental analysis of mechanical properties on AA6060 and 6061 aluminum alloys. Int. J. Eng. Res. Appl..

[19] Wagner M.F.X., Frint P. (2020). Formation of bulk-laminated materials by localized deformation during ECAP of an AA6060 aluminum alloy. MATEC Web Conf..

[20] Karon M., Kopysc A., Adamiak M., Konieczny J. (2016). Microstructure and mechanical properties of the annealed 6060 aluminium alloy processed by ECAP method. Arch. Comput. Mater. Sci. Surf. Eng..

[21] Yulinova A., Nickel D., Frint P., Lampke T. (2012). Electrochemical properties of AL-6060 alloy after industrial-scale ECAP. Mater. Sci..

[22] Chung C.S., Kim J.K., Kim H.K., Kim W.J. (2002). Improvement of high-cycle fatigue life in a 6061 Al alloy produced by equal channel angular pressing. Mater. Sci. Eng. A.

[23] Xu C., Furukawa M., Horita Z., Langdon T.G. (2005). Developing a superplastic forming capability in nanometals. Solid State Phenom..

[24] Horita Z., Furukawa M., Nemoto M., Barnes A.J., Langdon T.G. (2000). Superplastic forming at high strain rates after severe plastic deformation. Acta Mater..

[25] Islamgaliev R.K., Valiev R.Z., Burhanettin A. (2006). Enhanced Superplasticity of SPD-Produced Nanostructured Metallic Materials. Severe Plastic Deformation: Toward Bulk Production of Nanostructured Materials.

[26] Alawadhi M.Y., Sabbaghianrad S., Huang Y., Langdon T.G. (2021). Evaluating the paradox of strength and ductility in ultrafine-grained oxygen-free copper processed by ECAP at room temperature. Mater. Sci. Eng. A.

[27] Kamikawa N., Huang X., Tsuji N., Hansen N. (2009). Strengthening mechanisms in nanostructured high-purity aluminium deformed to high strain and annealed. Acta Mater..

[28] Tiža J., Kvačkaj T., Lupták M., Poór P. (2010). Study of Forces Changes in ECAP Process. Acta Metall. Slovaca Conf..

[29] (2017). Standard Test Method for Young’s Modulus, Tangent Modulus, and Chord Modulus.

[30] Harnicarova M., Valicek J., Kušnerová M., Kmec J., Palková Z., Kopal I., Krmela J., Panda A. (2019). Study of the influence of the structural grain size on the mechanical properties of technical materials. Mater. Werkst..

[31] Harničárová M., Valíček J., Kušnerová M., Palková Z., Kopal I., Borzan C., Kadnár M., Paulovič S. (2021). A New Method of Predicting the Structural and Mechanical Change of Materials during Extrusion by the Method of Multiple Plastic Deformations. Materials.

[32] Valíček J., Cep R., Rokosz K., Lukianowicz C., Kozak D., Zeleňák M., Koštial P., Hloch S., Harničárová M., Hlaváček P. (2012). New way to take control of a structural grain size in the formation of nanomaterials by extrusion. Mater. Werkst..

